# Disease-Associated Particulates and Joint Inflammation; Mechanistic Insights and Potential Therapeutic Targets

**DOI:** 10.3389/fimmu.2018.01145

**Published:** 2018-05-28

**Authors:** Olwyn R. Mahon, Aisling Dunne

**Affiliations:** School of Biochemistry and Immunology, School of Medicine, Trinity College Dublin, Trinity Biomedical Sciences Institute, The University of Dublin, Dublin, Ireland

**Keywords:** particulates, osteoarthritis, gout, calcium deposition disease, joint inflammation

## Abstract

It is now well established that intra-articular deposition of endogenous particulates, such as osteoarthritis-associated basic calcium phosphate crystals, gout-associated monosodium urate crystals, and calcium deposition disease-associated calcium pyrophosphate crystals, contributes to joint destruction through the production of cartilage-degrading enzymes and pro-inflammatory cytokines. Furthermore, exogenous wear-debris particles, generated from prosthetic implants, drive periprosthetic osteolysis which impacts on the longevity of total joint replacements. Over the last few years, significant insight has been gained into the mechanisms through which these particulates exert their effects. Not only has this increased our understanding of the pathological processes associated with crystal deposition but it has also led to the identification of a number of therapeutic targets to treat particulate-associated disease. In this review, we discuss recent developments regarding the cellular events triggered by joint-associated particulates, as well as future directions in therapy for particulate-related arthropathies.

## Introduction

Immune responses driven by microparticles are implicated in the pathogenesis of a number of inflammatory diseases, including atherosclerosis, silicosis, and asbestosis (1). In the joint, endogenous crystals, including uric acid and calcium-containing crystals, are formed as a result of dysregulated metabolic processes. These particulates are associated with gout, calcium pyrophosphate deposition (CPPD) disease, and osteoarthritis (OA), and a number of studies have demonstrated that they contribute to synovial inflammation, cartilage destruction, and subchondral bone remodeling (2–5). In cases of severe joint degeneration, total joint replacement (TJR) is the only remaining option to improve pain and ambulation in patients. However, the gradual wear of orthopedic implants over time results in the generation of particulate matter derived from various components of the prosthesis. The presence of these particles is associated with periprosthetic osteolysis which is characterized by inflammation and osteoclastic resorption of bone surrounding the implant (6, 7). Examination of the cell types and pathways activated following crystal deposition has shed light on the pathogenesis of crystal-related arthropathies and peri-implant inflammation, all of which are steadily becoming more prevalent as the lifespan of the general population increases. In this review, we discuss the molecular mechanisms underlying particulate matter-induced inflammatory and catabolic processes in the joint. We also highlight potential therapeutic targets for the treatment of particulate matter-induced inflammation.

## Monosodium Urate (MSU) Crystals and Gout

Gout is a chronic inflammatory disease characterized by high serum urate concentrations (>6.8 mg/dL) which in turn leads to MSU crystal deposition in joints and peri-articular tissues. While genetic and environmental factors contribute to the development of hyperuricaemia, the underexcretion of urate by specialized transporters in the gastrointestinal tract and kidney is considered a leading cause of elevated serum urate levels (8, 9). The precise mechanism(s) leading to MSU-crystal formation is not entirely understood; however, peripheral temperature, tissue pH, and synovial fluid components are all thought to play a contributory role when serum urate levels exceed maximal solubility. Clinical features of gout include acute inflammatory flares that tend to resolve spontaneously and, if untreated, can eventually advance to chronic gouty arthritis which is characterized by chronic inflammation and the formation of granulomatous lesions, known as tophi (10–12). Current treatments include urate lowering drugs, NSAID administration, and dietary modification; however, ongoing research into the mechanisms surrounding MSU crystal-induced cell activation has led to the identification of a number of novel targets that may also limit inflammation.

Monosodium urate crystals induce matrix metalloprotease (MMP) expression by chondrocytes (13), prostaglandin, and CCL2 production by fibroblasts and synoviocytes (14) and TGFβ1, IL-6, tumor necrosis factor (TNF)-α, and IL-8 secretion by monocytes (15–17). However, the acute joint inflammation observed during flare-ups is primarily driven by macrophage derived IL-1β, *via* activation of NOD-like receptor related protein 3 (NLRP3). MSU crystals are, therefore, classified as “danger signals” and, together with extracellular ATP and CPPD crystals, were the first endogenous activators of NLRP3 identified (18). Persistent NLRP3 activation has been linked to the pathogenesis of a number of inflammatory diseases and has been reviewed in detail elsewhere (19, 20). Briefly, activation of NLRP3 results in the assembly of a large multiprotein complex called the inflammasome which is involved in the processing of pro-IL-1 β and pro-IL-18 into their active forms. A “priming signal” (signal 1) upregulates expression of pro IL-1β/pro-IL-18 as well as components of the inflammasome complex and is mediated, for example, by TLR agonists. A second signal (Signal 2), caused by a disruption in cellular homeostasis, results in assembly of a complex comprised of NLRP3 oligomers and the adapter protein, ASC. Inactive pro-caspase-1 is recruited to the complex where it undergoes auto-activation and catalyzes the cleavage of pro-IL-1 β and pro-IL-18 into their mature forms which are secreted from the cell (21, 22). In the context of gout, a number of endogenous molecules, including free fatty acids, have been proposed to act as a priming signal, while MSU crystals act as signal 2 (23).

Crystal-induced inflammasome activation is linked to lysosomal rupture, ROS production and ATP driven P2X7-dependent pore formation (24). Potassium efflux is also involved in NLRP3 activation, and it has been proposed that fusion of MSU crystal-containing phagosomes with acidic lysosomes causes a massive release of sodium ions from the phagolysosome which is balanced by passive water influx. The accompanying drop in intracellular potassium concentration in turn drives NLRP3 assembly/activation (25). Targeting these aspects of NLRP3 inflammasome activation is currently being explored as a means of limiting the pathological effects of MSU crystals. For example, the ketone body, b-hydroxybutyrate, which specifically inhibits NLRP3 inflammasome activation *via* potassium efflux blockade, and the naturally derived antioxidant, sulforaphane, have been shown to attenuate MSU-induced responses in murine models of gout (26, 27). Furthermore, the anti-inflammatory phytochemical, caffeic acid phenyl ester, was shown to suppress MSU crystal-induced IL-1β production *in vivo* by directly blocking NLRP3/ASC interactions (28). It is worth noting that neutrophil-derived serine proteases are also capable of processing pro-IL-1β; therefore, direct neutralization of IL-1β may be of greater benefit in some patients (29). Indeed, canakinumab (anti-IL-1β) is recommended for the treatment of acute flares when other anti-inflammatory drugs are ineffective.

Targeting upstream of NLRP3 activation is also a possibility and recent studies have provided insight into the cellular events triggered upon binding of MSU crystals to the cell membrane. Barabe et al. demonstrated that MSU crystals bind directly to the surface Fc receptor, FcgRIII, in human neutrophils (30) while Desaulniers et al. demonstrated that MSU crystals trigger activation of the downstream signaling molecule, Syk (31). It was subsequently demonstrated that Syk is a substrate of conventional PKCs, which are activated in a Src kinase-dependant manner (32). Phosphorylation of Syk by PKC facilitates the interaction of Syk with PI3 kinase driving subsequent phagocytosis of MSU crystals (33). These effects may, however, be cell-type specific as it was later demonstrated that Syk is activated by MSU crystals in a receptor-independent manner in dendritic cells, *via* a process known as membrane affinity-triggered signaling (MATS) (34). This involves direct binding of particulate matter to the cell membrane which results in clustering of lipid rafts and aggregation of proteins that are partitioned into membrane lipids. Atomic force microscopy confirmed the direct interaction of MSU crystals with cell-surface lipids, particularly cholesterol. This in turn leads to aggregation of immunoreceptor tyrosine-based activation motif-containing molecules which then recruit and activate Syk (35). Inhibition of Src family kinases prevented MSU-induced Syk phosphorylation while PI3 kinase was found to be activated downstream of Syk; however, unlike neutrophils, surface receptors were not required for this interaction as treatment with pronase had no effect on MSU uptake (34).

While Syk inhibitors can attenuate MSU-induced IL-1β production *in vitro* (36), it remains to be seen whether direct intra-articular administration of Syk inhibitors can ameliorate the effects of MSU crystals in a clinical setting. Indeed a number of kinases are likely to be activated downstream of Syk. Of note, the broad spectrum tyrosine kinase inhibitor, imatinib mesylate, was recently shown to suppress MSU crystal-induced synovial inflammation in an acute gouty arthritis model (37).

Neutrophil necroptosis, a regulated form of necrosis, has also been implicated in MSU-induced responses (38, 39). In contrast to apoptosis, necroptosis occurs independently of caspase activation and may exacerbate inflammation *via* the release of additional danger signals (40). Receptor interacting protein kinase-1, RIP3, and mixed lineage kinase domain-like (MLKL), play key roles in necroptosis. Targeting RIP1 and MLKL was shown to suppress MSU-induced cell death *in vitro* while MLKL-deficient mice lacked tophus formation in a gouty arthritis model, providing further confirmation of the involvement of the RIP1-RIP3-MLKL axis (38). Neutrophil extracellular traps (NETs) are formed during MSU-induced necroptosis but whether this is an active process or a passive consequence of unregulated necrosis has yet to be determined and is discussed in more detail in Ref. (41). While it was previously reported that ROS production is required for macroscopic aggregation of NETs and crystals (42), a recent study by Chatfield et al., involving direct quantitation of key aspects of NET formation, has demonstrated that ROS production is dispensable for MSU-induced NET formation in human neutrophils (43). Furthermore, neutrophils from patients with a functional NOX2 deficiency are capable of producing NETs in response to MSU crystal stimulation (44).

**Figure 1:**
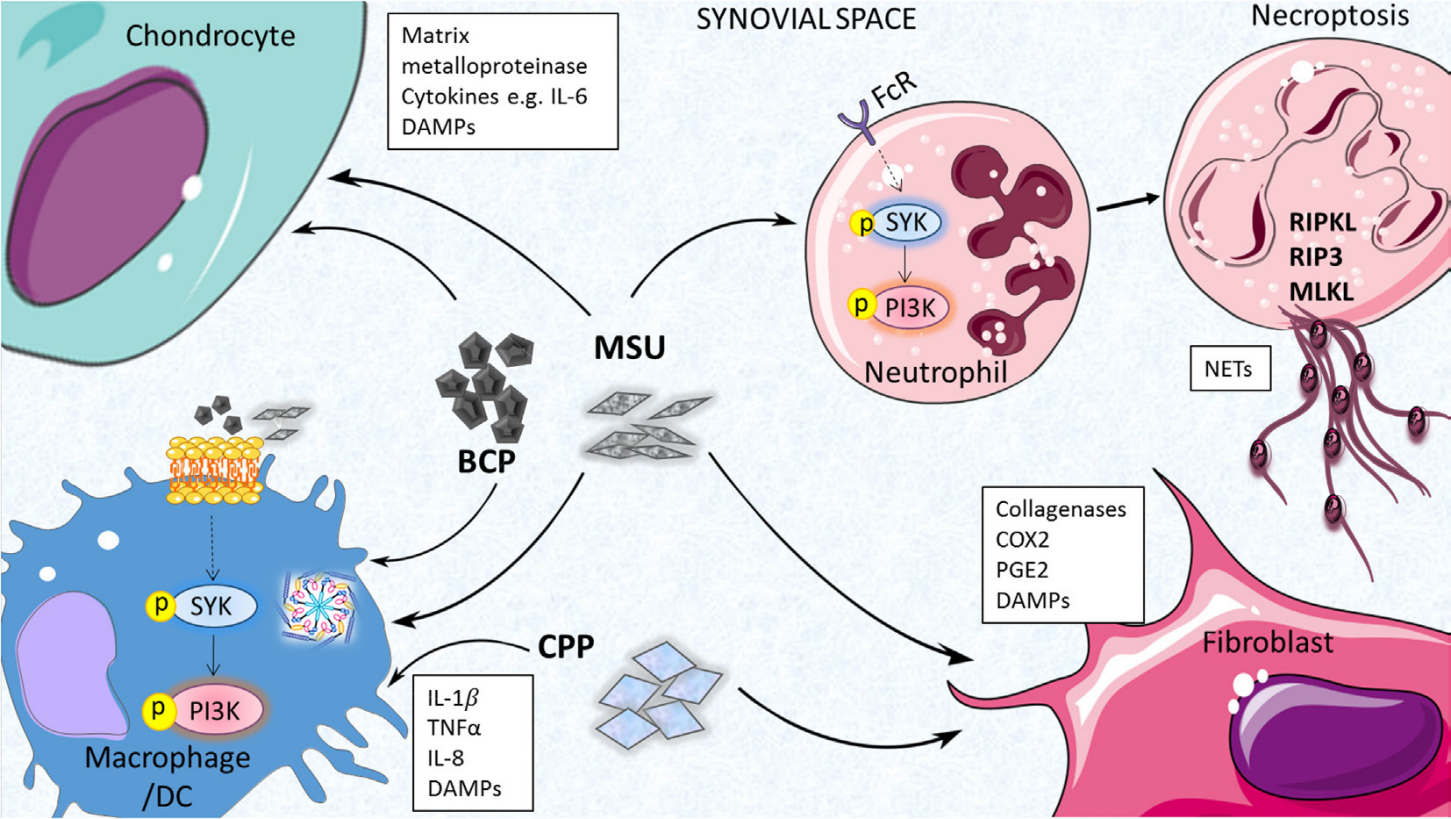
Inflammatory processes driven by endogenous joint-associates particulates. Monosodium urate (MSU), basic calcium phosphate (BCP) and calcium pyrophosphate (CPP) crystals can act on a number of cell types in the joint to drive pro-inflammatory cytokine production, matrix metalloprotease (MMP) expression, and release of additional damage-associated molecular patterns (DAMPs) that contribute further to joint inflammation and cartilage destruction. Inflammasome activation, membrane affinity-triggered signaling, and neutrophil-mediated neutrophil extracellular trap (NET) formation have been implicated in these responses.

Neutrophil extracellular traps have been detected in synovial fluid from acutely inflamed joints of gouty patients and also surrounding crystals in non-inflamed tophi of chronic gout patients (43). NETs are released by neutrophils to trap and kill invading microbes during infection and neutrophil granule proteases have been shown to activate cytokines such as IL-1α and IL-33 (45); however, it has been suggested that they may actually play a pro-resolving role in acute gout *via* degradation of pro-inflammatory cytokines and chemokines (42). Further study is clearly required to fully appreciate the role of neutrophil-mediated processes in the pathogenesis of gout. The cell types and mediators of MSU-induced inflammation are highlighted in Figure 1.

## Calcium-Containing Crystals in OA and CPPD Disease

Basic calcium phosphate (BCP) crystals, of which the hydroxyapatite (HA) form is most prevalent, are found in 70% of total OA cases where their concentration closely correlates with the extent of cartilage degradation and lesion severity (46, 47). They are thought to form as a result of dysregulated ossification processes (48) and, given that crystal deposition does not occur in healthy cartilage, it is becoming more widely accepted that cartilage calcification plays a pathogenic role in OA (3). Like MSU crystals, BCP crystals are now considered a “danger signal” as they can activate a number of cell types and contribute to joint degeneration through the production of cartilage-degrading proteases and pro-inflammatory mediators. Early studies demonstrated that BCP crystals drive MMP and inflammatory gene expression in fibroblasts (49, 50), while more recent studies have focused on their effects on macrophages, chondrocytes, and osteoclasts (51–54). Macrophage derived-IL-1β has been given a lot of consideration in the context of BCP crystals and OA, and while BCP crystals induce potent IL-1β production *in vitro*, reports from *in vivo* studies have been conflicting (55, 56). Indeed, Nasi et al. recently demonstrated that neither IL-1α nor IL-1β mediate the pathology seen in the murine menisectomy model of OA (57), a finding that may explain the lack of efficacy of IL-1 inhibitors in human OA. Based on these studies, focus has shifted to other potential targets including IL-6 which is produced by chondrocytes in response to BCP stimulation and acts in an autocrine manner to promote calcium-containing crystal formation and upregulation of genes involved in calcification (51).

We recently demonstrated that, like MSU crystals, BCP crystals also activate, Syk, and its downstream interacting partner, PI3K, in primary macrophages, and that pharmacological inhibition of these kinases leads to reduced MMP expression and pro-inflammatory cytokine production (53, 54). Whether Syk and/or PI3K activity is heightened in OA joints is yet to be determined, however, targeting membrane-proximal events induced by BCP crystals is a therapeutic option worth exploring. We have also demonstrated that both MSU and BCP crystals may contribute to bone erosion *via* inhibition of anti-osteoclastogenic cytokine signaling (58); therefore, targeting synovial inflammation induced by crystal deposition may suppress early destructive processes, while targeting the osteoclastogenic effects of crystals may suppress excessive bone remodeling. Indeed, preventing the actual intra-articular deposition of calcium crystals, as recently demonstrated by Nasi et al. using sodium thiosulphate, could limit inflammatory responses at the outset (59).

Pathological calcification is also a feature of CPPD disease. Acute CPPD disease is caused by the deposition of calcium pyrophosphate (CPP) crystals and is accompanied by symptoms overlapping with acute gouty arthritis, hence it is often referred to as pseudogout [reviewed in Ref. (5)]. A local excess of pyrophosphate (PPi) has been observed in the cartilage of affected patients and it is believed that high levels of extracellular PPi complex with calcium in the chondrocyte pericellular matrix to form CPP crystals. Like MSU crystals, CPPD crystals are phagocytosed by macrophages leading to NLRP3 dependent IL-1β processing and secretion and there is some evidence to suggest that anti-IL-1 therapies may be of benefit to patients (18). CPPD crystals have also been shown to drive the formation of NETs *in vitro* (60); however, whether this contributes to joint inflammation or plays a pro-resolving role *in vivo* has yet to be determined. Cartilage destruction is driven largely by the action of MMPs produced by synovial fibroblasts and chondrocytes and in patients with severe disease, joint replacement may be required. One potential therapeutic target for CPPD disease that has received a lot of attention of late is the multipass membrane protein, ANK (protein product of the progressive ankylosis gene), which regulates levels of inorganic phosphate. A gain of function mutation in human ANK is associated with familial cases of CPPD while expression of the protein was found to be increased in the cartilage of patients with sporadic CPPD disease (61). Given the potential link between crystal deposition and ANK activity, modulation of this pathway may be of benefit for the treatment, not just of pseudogout, but other arthopathies involving pathological CPP deposition.

## Wear-Debris Particles and Periprosthetic Osteolysis

Total joint replacement is a highly successful procedure used to alleviate pain and restore function in individuals suffering from end-stage joint disease. Implants are commonly composed of a metal, e.g., titanium or ultra-high-molecular-weight polyethylene, and are coated with a bioceramic, such as HA, to enhance integration with surrounding bone. The implant is then typically fixed into place with poly-methyl methacrylate (PMMA) bone cement. While excellent patient outcomes are associated with this procedure, revision surgeries are often required due to the limited lifespan of orthopedic devices. The gradual wear and tear of implants over time results in the continuous generation of wear-debris particles from the articulating surface of the prosthesis (62). Due to their insoluble nature, most wear particles are resistant to digestion by macrophages and thus, a chronically activated macrophage population persists in the joint (63). This then culminates in periprosthetic osteolysis which is characterized by the osteoclastic resorption and degradation of bone surrounding the implant, a process that ultimately leads to aseptic implant loosening (64–66).

**Figure 2:**
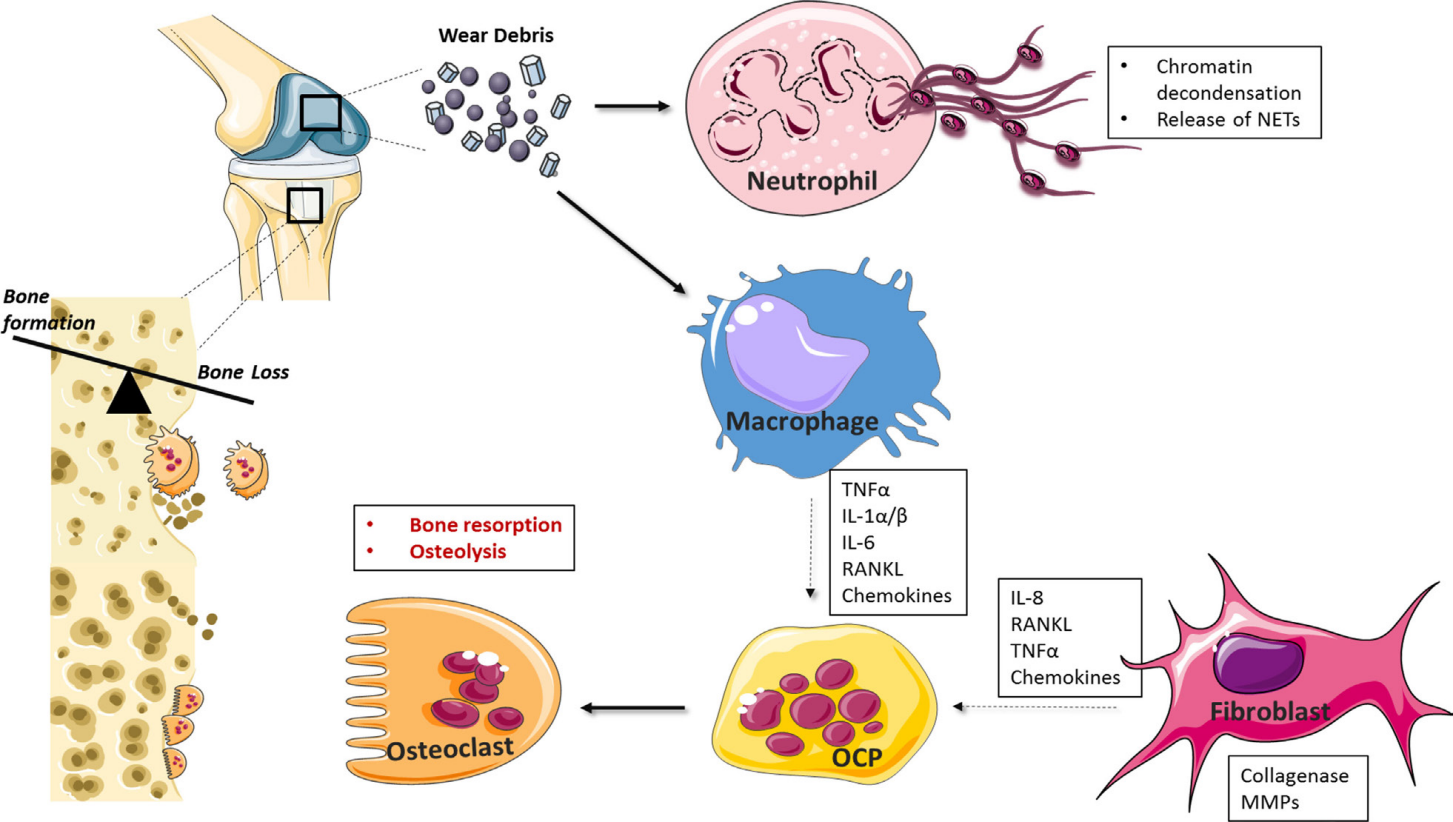
Cellular mediators of periprosthetic osteolysis. Wear-debris particles generated from prostheses activate resident and infiltrating macrophages, fibroblasts, and neutrophils at the site of implantation. Neutrophil recognition of biomaterials drives neutrophil extracellular trap (NET) formation while macrophage and fibroblast-induced chemokine production facilitates further leukocyte recruitment to the implant interface. Cytokines, such as tumor necrosis factor (TNF)-α and IL-1, drive inflammation and, together with RANKL, can induce the differentiation of osteoclast precursor cells (OCPs) into activated bone resorbing cells. Matrix metalloprotease (MMPs) and collagenases contribute further to catabolic processes and aseptic implant loosening.

A number of cell types, including osteoclasts, fibroblasts, and osteoblasts, have been implicated in wear-debris induced osteolysis (Figure 2) (67, 68). More recently, various types of nanoparticles have been shown to act on neutrophils, inducing the formation of NETs (69–73). For example, Vitkov et al. demonstrated that PMN cells incubated with sandblasted large-grit acid etched (SLA) implants undergo histone citrullination, nuclear swelling, and chromatin alterations (70), while Muñoz et al. demonstrated that carbon and polystyrene nanopowders increase NET formation in a size-dependent manner (72). Macrophages play a key role in osteolysis where particle uptake culminates in a chronic inflammatory state mediated by cytokines, such as IL-1, IL-6, TNF-α, and RANKL, which can also contribute to osteoclast differentiation and thus exacerbate bone resorption (74–78). Chemokine production is also central to wear-debris particle-induced inflammatory responses (79). For example, MIF, MCP1, MIP 1α RANTES, and IL-8 levels are elevated in macrophages in response to PMMA and titanium particles (80–82), while fibroblasts produce elevated levels of MCP-1 after exposure to titanium and PMMA particles (83).

Like other disease-associated particulates, it is likely that wear-debris particles are binding to scavenger receptors on the cell surface or are inducing MATS. We have recently demonstrated that this is the case for HA and PMMA particles and that inhibition of Syk prevented wear particle-induced M1 macrophage polarization *in vitro* (84). Indeed, modulation of macrophage phenotype has been suggested as a potential therapeutic avenue for periprosthetic osteolysis and administration of the M2 polarizing cytokine, IL-4, has shown some efficacy, at least in murine models (85, 86). There are, however, no drugs specifically approved for the prevention of periprosthetic osteolysis. Bisphosphonates have been shown to inhibit enzymes in the mevalonate pathway which results in apoptosis of osteoclasts (68). They are commonly used for metabolic bone diseases (87) and several experimental studies have demonstrated a significant decrease in osteolysis/bone resorption after treatment with these compounds (88, 89). Modulating pro-inflammatory cytokine production may also prove beneficial for osteolysis patients assuming inflammation is targeted prior to extensive bone damage. Bortezomib (Bzb) is a reversible 26S proteasome inhibitor currently approved for the treatment of relapsed/refractory multiple myeloma (90). As well as antitumor effects, it has been shown to limit inflammation and bone resorption in arthritis models (91, 92). Bzb inhibits NF-κB, a master regulator of inflammation, by blocking degradation of the NF-κB inhibitor, IkB. Mao et al. recently demonstrated that Bzb can inhibit titanium particle-induced inflammation in murine macrophages, a finding that warrants further *in vivo* study as it may have implications for the treatment of periprosthetic inflammation.

Finally, autophagy is associated with a number of physiological processes including basal inhibition of inflammation (93). Crişan et al. recently demonstrated that uric acid can inhibit autophagy, a finding that has implications not just for gout but other diseases associated with elevated uric acid levels including cancer and type 2 diabetes (94). Indeed, titanium particles can downregulate the expression of osteocyte-derived IFNβ in an autophagy-dependent manner (95). IFNβ is a negative regulator of osteoclastogenesis (96); therefore, therapeutic interventions to boost autophagy, for example with mTOR inhibitors or naturally derived autophagy inducers such as trehalose (97), may be of benefit for both gout and osteolysis patients.

## Conclusion

While much progress has been made in elucidating the events contributing to crystal deposition diseases, the complex nature of these conditions has hampered the development of effective treatments. Urate-lowering drugs have proven efficacy in gout; however; in the case of OA, and in some instances, CPPD disease, TJR remains the only option for patients with significant joint destruction. This in itself is associated with the added complication of periprosthetic osteolysis and potential implant failure. The development of more durable biomaterials with low immunogenicity may prevent the occurrence of periprosthetic inflammation, while the identification of successful disease-modifying drugs for severe arthropathies may relinquish the need for joint replacement in the first instance. Therefore, gaining a better understanding of the inflammatory and destructive processes driven by disease-associated particulates, coupled with advances in disease monitoring technology, will be of huge benefit for the development of future prevention/treatment strategies.

## Author Contributions

AD conceived and wrote the paper with contribution from OM.

## Conflict of Interest Statement

The authors declare that the research was conducted in the absence of any commercial or financial relationships that could be construed as a potential conflict of interest.
